# Human alpha-herpesvirus 1 (HSV-1) viral replication and reactivation from latency are expedited by the glucocorticoid receptor

**DOI:** 10.1128/jvi.00303-25

**Published:** 2025-03-27

**Authors:** Clinton Jones

**Affiliations:** 1 Department of Veterinary Pathobiology, Oklahoma State University, College of Veterinary Medicine 70729, Stillwater, Oklahoma, USA; Universiteit Gent, Merelbeke, Belgium

**Keywords:** HSV-1, stress response, glucocorticoid receptor, reactivation from latency

## Abstract

Acute human alpha-herpesvirus 1 (HSV-1) infection leads to infection of neurons within trigeminal ganglia (TG), brainstem, and other regions of the central nervous system. Lytic cycle viral gene expression is subsequently silenced, a subset of neurons survive infection, and life-long latency is established. In contrast to lytic infection, the latency-associated transcript (LAT) is the only viral gene product abundantly expressed in latently infected neurons. Stress (acute or chronic), UV light, or heat stress increases the incidence of reactivation from latency in humans and mouse models of infection. Ironically, these divergent reactivation stimuli activate the glucocorticoid receptor (GR). Recent studies revealed GR and Krüppel-like factors (KLF), KLF4 or KLF15 for example, cooperatively transactivate the infected cell protein 0 (ICP0) promoter and cis-regulatory motifs that activate ICP4 and ICP27 promoter activity. GR and KLF4 are “pioneer transcription factors” that specifically bind DNA even when it exists as heterochromatin; consequently, chromatin is remodeled, and transcription is activated. Conversely, a VP16 cis-regulatory motif is transactivated by GR and Slug but not KLF family members. Female mice that express a GR containing a serine → alanine mutation at position 229 (GR^S229A^) shed significantly lower HSV-1 levels compared with age-matched male GR^S229A^ mice or wild-type parental C57BL/6 mice during reactivation from latency. These observations imply GR and stress-induced cellular transcription factors play an important role during reactivation from latency by activating key viral promoters. GR activation may also enhance virus spread by impairing immune and inflammatory responses.

## HSV-1 LATENCY–REACTIVATION CYCLE IS ESSENTIAL FOR RECURRENT DISEASE

During acute HSV-1 infection of craniofacial mucosal surfaces, HSV-1 infects neurons in the trigeminal ganglia (TG), brainstem, and additional regions of the central nervous system (1–3). Lytic cycle viral gene expression was reported in TG of latently infected mice (4, 5). A subset of infected neurons survives, the viral genome is circularized, and most of the genome exists as silent chromatin (6–8). Viral protein expression in neurons is not readily detected during latency. Moreover, virus shedding is not routinely detected in ocular or nasal swabs. The HSV-1 latency–reactivation cycle is operationally divided into three steps: establishment, maintenance, and reactivation (9, 10). The ability of HSV-1 to successfully reactivate from latency leads to virus shedding, transmission to other people, and recurrent disease. Approximately 400,000 individuals in the United States suffer from HSV-1 ocular disease, including herpetic stromal keratitis (11, 12) and blindness (13). HSV-induced encephalitis is the most common cause of sporadic and fatal encephalitis, and most cases are the result of reactivation from latency (14, 15). Identifying cellular and viral factors that stimulate reactivation from latency is important because there are no HSV-1 or HSV-2 vaccines, and acyclovir does not effectively reduce the incidence of reactivation from latency or recurrent disease.

In contrast to productive infection, the latency-associated transcript (LAT) is the only viral transcript abundantly expressed during latency. The LAT locus encodes a long-coding RNA, six micro-RNAs, two small non-coding RNAs that are not micro-RNAs, and three transcripts antisense to LAT, reviewed in reference 9. LAT-encoded products impair apoptosis (16–18), productive infection (19, 20), and promote neuronal differentiation (18, 21). LAT expression also promotes maintenance of latency and periodic reactivation from latency (22). It is not clear whether LAT encodes a function that regulates the latency–reactivation expression.

## DIVERGENT REACTIVATION STIMULI ACTIVATE THE GLUCOCORTICOID RECEPTOR (GR)

Stress (acute, episodic acute, or chronic), heat stress, or UV light increase the incidence of reactivation from latency in humans (10, 23–29). Furthermore, these stimuli trigger reactivation from latency in mouse models of latency (30–32). Heat stress increases cortisol and activates the glucocorticoid receptor (GR) (33). UV light also triggers GR phosphorylation and transcriptional activation via ligand-independent mechanisms (34, 35). UVB and UVC light, but not UVA, increase cortisol production in human skin cultures, and UVB light increases corticosteroid levels in C57BL/6 mice (36, 37). GR activation has the potential to directly stimulate HSV-1 gene expression in latently infected neurons because ~50% of TG neurons express GR (38).

**Fig 1:**
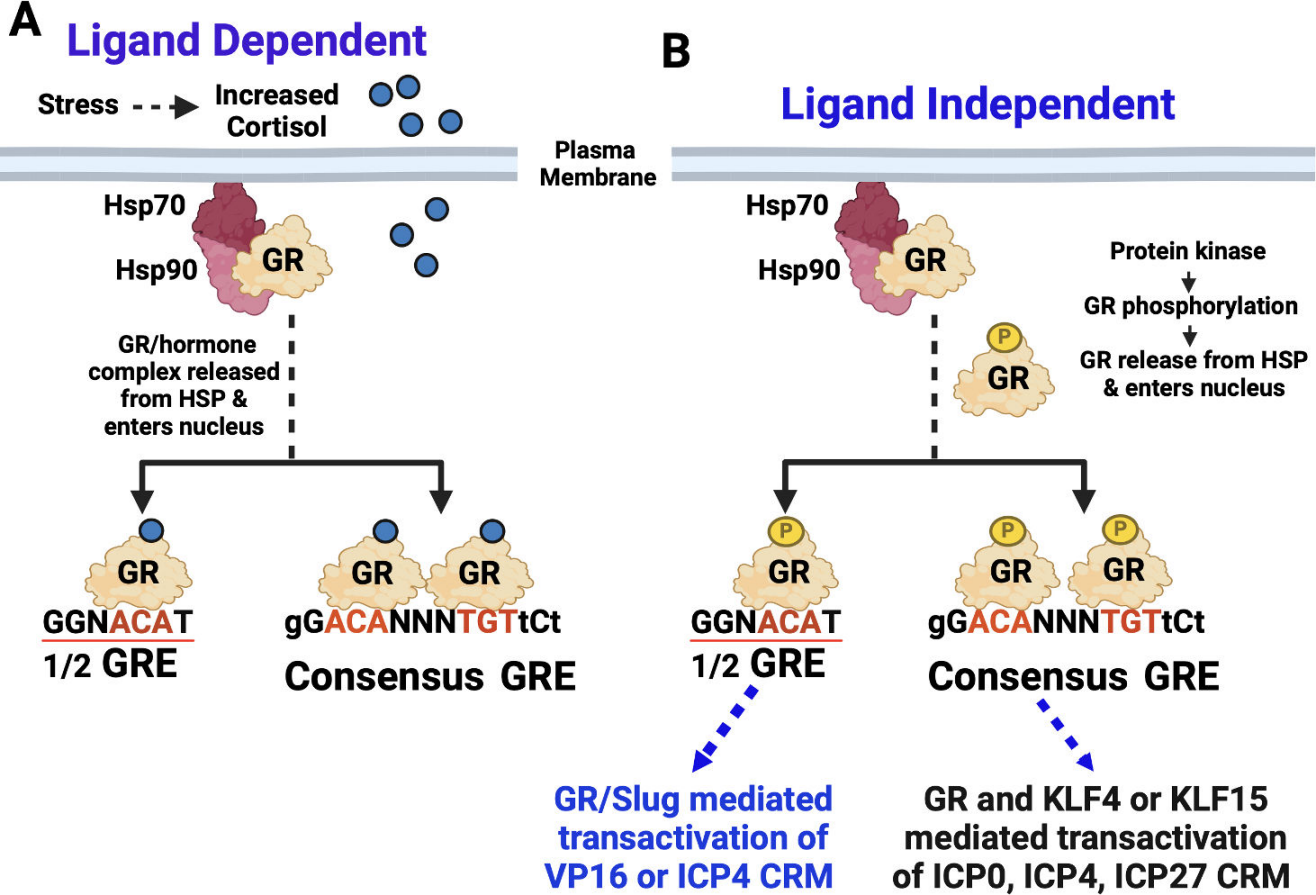
Activation of GR by corticosteroids and protein kinases. (A) Schematic of key events necessary for GR activation by increased glucocorticoids secreted via the hypothalamic pituitary axis (HPA). Red nucleotides in the GRE are essential, capital letters are well-conserved nucleotides, small letters are flexible, and N can be any nucleotide. A GR–hormone dimer specifically binds to a consensus GRE. A GR–hormone homodimer can also bind certain 1/2 GREs and stimulate transcription. The 1/2 GRE is a common consensus that can be transactivated. (B) Specific protein kinases can phosphorylate GR, which releases GR from the HSP complex (phosphorylated GR is denoted as GR-P). A phosphorylated GR dimer or GR monomer enters the nucleus, binds a consensus GRE or 1/2 GRE, respectively, and activates transcription.

Generally, stressful stimuli trigger cortisol secretion via the hypothalamic-pituitary adrenocortical (HPA) axis, reviewed in reference 39. Consequently, cortisol diffuses into cells and binds GR (Fig. 1A). The GR–hormone complex is released from the heat shock protein (HSP) complex and rapidly enters the nucleus. A GR-hormone dimer binds to a consensus GR response element (GRE), remodels chromatin, and stimulates transcription via a ligand-dependent mechanism. A GR monomer bound to cortisol can also bind a 1/2 GR. GR phosphorylation by specific protein kinases culminates in GR nuclear localization and is referred to as ligand-independent GR activation (Fig. 1B) (34, 40). These steps do not require *de novo* protein synthesis, which allows rapid responses following stressful stimuli, and are commonly referred to as genomic effects (41, 42). Non-genomic effects involve non-specific GR interactions with cell membranes that activate numerous signaling pathways. These signaling pathways, including protein kinase A (PKA), PKC, AMP-activated protein kinase, Rho kinase, nitric oxide signaling, extracellular signal-regulated kinase 1 (ERK1), ERK2, mitogen-activated protein kinase, JNK, Src, and phosphatidylinositol 3-kinase/Akt signaling axis, reviewed in reference 43. Genomic effects of GR appear to be very similar in most cells, including neurons, whereas non-genomic GR signaling may have significant differences in neurons. However, these differences are not well established.

GR phosphorylation at the murine serine 134 (34) is important for ligand-independent GR activation (34) (Fig. 1). GR serine 134 is phosphorylated by mitogen-associated protein kinases, cyclin-dependent kinases, glycogen synthase kinase 3 beta, and possibly other unknown protein kinases (34). Glucose starvation, oxidative stress, UV irradiation, and osmotic shock lead to hyperphosphorylation of GR serine 134. A monomer GR can also stimulate transcription by binding to certain 1/2 GREs (44, 45). Phosphorylation of human GR serine 211 (mouse serine 229) is necessary for optimal GR-mediated transcriptional activation, reviewed in reference 38. Mutating serine 211 of the human GR induces conformational changes in GR activation function region 1, which correlates with reduced transactivation of promoters containing GREs (39). Conversely, GR that is not phosphorylated at serine 211 does not influence GR-dependent trans-repression (46). Although the details of ligand-independent GR activation are not fully understood, unliganded GR activation stimulates a different subset of cellular genes when compared with ligand-dependent GR activation (34), including the tumor suppressor Breast Cancer Gene 1 (47).

Increased corticosteroids and GR activation have potent anti-inflammatory and immune-suppressive effects, in part by inactivating transcription factors (AP-1 and NF-κB) that stimulate expression of inflammatory cytokines, reviewed in reference 48. Corticosteroids can also induce apoptosis, including certain lymphocyte subsets, which enhances immune suppression (49, 50). Although anti-inflammatory and immune-suppressive properties of GR are important for viral spread, the ability of GR and stress-induced transcription factors to trigger viral gene expression is predicted to drive early stages of reactivation.

## GR ACTIVATION STIMULATES HERPESVIRUS REPLICATION AND REACTIVATION FROM LATENCY

Stress, as mimicked by the synthetic corticosteroid hormone dexamethasone (DEX), consistently initiates bovine alpha-herpesvirus 1 (BoHV-1) reactivation from latency in latently infected calves or rabbits (51–53). Synthetic corticosteroids also stimulate reactivation from latency in beagles latently infected with canine herpesvirus 1 (54). These studies support the premise that stress and GR activation increase the incidence of reactivation from latency in their natural host.

Several studies demonstrated stress stimulates HSV-1 replication in cultured cells and reactivation from latency. For example, treatment of human gingival fibroblasts with glucocorticoids enhances HSV-1 replication (55). Furthermore, DEX stimulates HSV-1 productive infection in mouse neuroblastoma cells (Neuro-2A) (56) and accelerates virus shedding during explanted-induced reactivation (57, 58). Conversely, CORT-108297, a GR-specific antagonist, impairs virus shedding during explant-induced reactivation (57). Heat stress reproducibly initiates reactivation from latency in mouse models of infection (32). Notably, inhibiting cortisol production impairs heat stress-induced HSV-1 reactivation (59) in part because heat stress increases cortisol and activates GR (33). An intravenous injection of cyclophosphamide, which suppresses immune response, followed by an intravenous injection of DEX 24 h later consistently induces reactivation from latency in a mouse model of infection (30).

While TG is a primary site for latency, HSV-1 but not HSV-2 DNA is also detected in autonomic and sensory ganglia of the head and neck of human cadavers (60). Neurons from adult female Swiss Webster mice were prepared from TG, sympathetic superior cervical ganglia, and parasympathetic ciliary ganglia and subsequently infected with HSV-1 or HSV-2. To establish a quiescent/latent infection, the antiviral drug acyclovir was added to establish and maintain quiescent infections. Following removal of acyclovir, corticosterone induced HSV-1 reactivation in purified sympathetic neurons and HSV-2 reactivation in sensory and sympathetic neurons (61). If primary sympathetic neurons are prepared from postnatal superior cervical ganglia and then infected with HSV-1 infection, adding the antiviral WAY-150138 compound blocks viral replication (62). Removal of WAY-150138 led to viral replication, and addition of DEX to these cultures dramatically increased viral reactivation. Epinephrine, but not corticosterone, induced HSV-1 reactivation using purified sensory neurons from TG (61). These interesting studies provided evidence that certain purified neuronal subtypes respond differently to corticosterone relative to epinephrine. Currently, it is unclear why DEX consistently accelerates TG explant-induced reactivation from latency.

## IDENTIFICATION OF STRESS-INDUCED TRANSCRIPTION FACTORS DURING EARLY STAGES OF REACTIVATION FROM LATENCY

Viral regulatory proteins are not abundantly expressed during latency, indicating that stress-induced cellular transcription factors stimulate viral gene expression during early stages of reactivation. Stress-induced transcription factors and signaling pathways activated during reactivation from latency were identified using microarrays and bulk-RNA transcriptomic studies of TG during latency versus reactivation from latency in calves latently infected with BoHV-1 (63, 64). Interestingly, increased expression of 4 Krüppel-like factors (KLF) were identified in bovine TG during DEX-induced reactivation from latency (63): KLF4, KLF6, KLF15, and promyelocytic leukemia zinc finger (PLZF). PLZF RNA expression is 16-fold higher at 3 h after DEX treatment relative to latently infected calves. KLF15, SPDEF (SAM pointed domain-containing Ets), Slug (an E-box binding protein), and GR are also expressed in more TG neurons from mouse TG explants treated with DEX versus no DEX treatment (65), indicating the stress response is similar in bovine and mice.

## KLF AND SP FAMILY MEMBERS COMPRISE A SUPER-FAMILY OF TRANSCRIPTION FACTORS THAT INTERACT WITH GC-RICH DNA

The 18 KLF family members and nine specificity protein (Sp) family members form a super-family of transcription factors, reviewed in references 66, 67). Members of this super-family bind GC-rich motifs, CA-, or CC-rich motifs. Sp1 and KLF family members contain a DNA-binding domain that has ~65% sequence identity and three tandem Cys_2_His_2_ zinc-finger motifs at the C-terminus. The zinc-finger motifs mediate protein–protein interactions and DNA-binding specificity. The amino-terminal regions are variable and mediate transcriptional activation or repression. As expected, these proteins contain nuclear localization sequences that are adjacent to or overlap the Cys_2_His_2_ zinc-finger motifs. Since the HSV-1 genome is GC-rich (68), KLF and Sp transcription factors are predicted to regulate viral gene expression.

Sp1 and Sp3 are expressed in most cells and surprisingly exhibit important differences. HSV-1 latently infected male C57Bl/6 mice contained significantly more Sp1+ TG neurons during latency relative to 8 h after DEX treatment of TG explants (69). Conversely, Sp3 expression was detected in more neurons of male and female mice when TG explants were treated with DEX for 8 h relative to TG from latently infected mice. Sp1 overexpression can also induce apoptosis (46, 70), whereas other studies concluded Sp1 and Sp3 impair apoptosis (71). Sp1 stimulates HSV-1 replication by interacting with and transactivating IE promoters (ICP4, ICP22, and ICP47) (69, 72, 73), and Sp1 is phosphorylated during HSV-1 infection (74). Suppressing Sp1 expression in cultured mouse neuronal cells (Neuro-2A) with a specific siRNA or mithramycin A, an antibiotic that selectively binds to GC-rich DNA, significantly inhibits HSV-1 replication, confirming that G-C rich transcription factors play key roles during viral replication (69). Interestingly, apoptosis signaling pathways were reported to accelerate reactivation from latency (58, 75). However, cell death *per se* is not essential for reactivation from latency because neurons prepared from superior cervical ganglia of Bax-knockout mice, which are resistant to apoptosis, and wt mice reactivate from latency with the same efficiency (62).

## TRANSACTIVATION OF HSV-1 PROMOTERS BY GR AND STRESS-INDUCED TRANSCRIPTION FACTORS

Initial studies tested whether GR and stress-induced transcription factors transactivate the infected cell protein 0 (ICP0), ICP4, and viral protein 16 (VP16) promoters and/or cis-regulatory motifs (CRM). The rationale for choosing these viral promoters is ICP0, ICP4, or VP16 protein expression induces reactivation from latency in primary cultures of latently infected TG cells (76). All α-herpesvirinae subfamily members encode ICP0, ICP4, and VP16. ICP0 is a promiscuous transactivator of all viral promoters, impairs innate immune responses, and dissolves anti-viral promyelocytic leukemia nuclear bodies (77–79). ICP4 binds ~100 consensus binding sites in the HSV-1 genome (80) and recruits the TATA box-binding protein and RNA pol II transcription factor IIB to activate early and late viral promoters (81). Thus, ICP4 is essential for productive infection (82). The tegument protein, VP16, specifically stimulates IE transcription via interactions with two cellular proteins (Oct1 and host cellular factor 1) (83–85). VP16, ICP0, and ICP4 proteins are readily detected in TG neurons within 8 h after TG explants are incubated with media, stripped FBS, and DEX (57, 86). An ICP27 CRM was also examined because this gene is essential for productive infection (87).

### Transactivation of ICP0 promoter by GR and KLF family members

**Fig 2:**
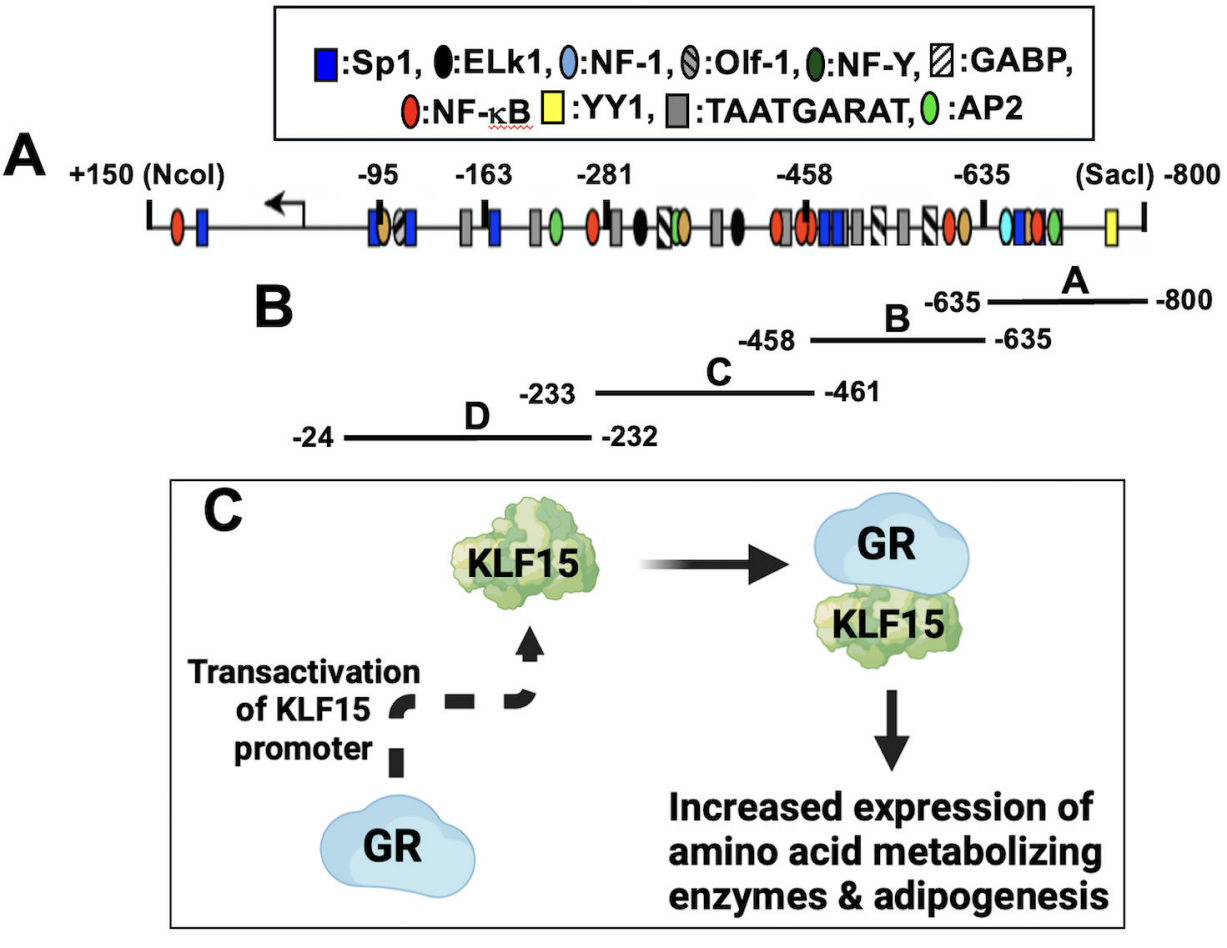
Schematic of ICP0 promoter, ICP0 CRMs, and feed-forward transcription loop that drives ICP0 promoter activity. (A) Schematic of ICP0 promoter and location of cellular transcription factor binding sites relative to the start site of ICP0 mRNA (arrow). (B) Four ICP0 CRM fragments (A–D) were cloned upstream of the pGL4.24[luc2/minP] firefly luciferase reporter plasmid (Promega: Madison, WI) as previously described (88). (C) Schematic of GR and KLF15 feed-forward transcription loop. GR stimulates KLF15 expression, and subsequently, a stable complex comprised of GR/KLF15 synergistically transactivates promoters that drive expression of amino acid metabolizing enzymes and adipogenesis.

GR and KLF15 cooperatively transactivate the full-length ICP0 promoter (−850 to +150; Fig. 2A) when DEX is added, indicating transactivation occurs via a ligand-dependent mechanism (56). Although GR and KLF4 or KLF6 significantly stimulates ICP0 promoter activity, the effects were dramatically lower compared to GR and KLF15. Deletion mutants of the full-length ICP0 promoter led to incremental reduction of GR and KLF15-mediated transactivation. Mutating the five putative 1/2 GRE-like motifs in the full-length ICP0 promoter contains only reduced GR, KLF15, and DEX-mediated transactivation by twofold. Chromatin immunoprecipitation (ChIP) studies revealed that GR and KLF15 occupy ICP0 promoter sequences in transfected cells or productively infected cells.

Four CRM fragments spanning the full-length ICP0 promoter (Fig. 2B) were inserted upstream of a minimal promoter in a luciferase reporter plasmid and then tested for transactivation by GR and KLF15 (88). Constructs containing CRM A, B, or D were cooperatively transactivated by GR and KLF15 in Neuro-2A or Vero cells. DEX treatment of transfected cultures did not significantly increase promoter activity. Silencing KLF15 expression significantly reduces HSV-1 replication in cultured cells (89), confirming that KLF15 expression is important for viral replication and gene expression. Additional studies tested whether GR and Sp1 or Sp3 transactivate the ICP0 promoter because consensus Sp1 binding sites, not the 1/2 GREs, are essential for GR- and KLF15-mediated transactivation. We focused on Sp1 and Sp3 because these proteins are expressed in most cells (90).

GR and Sp1 or Sp3 significantly transactivate the ICP0 CRM A, B, and D fragments but not CRM C, which is consistent with the results observed with GR and KLF15 (91). Mutating Sp1 binding sites in the ICP0 A, B, or D CRM fragments significantly reduced GR and Sp1- or Sp3-mediated transactivation. Co-immunoprecipitation studies revealed GR and Sp1 interact, suggesting this interaction is important for transcriptional activation (92).

Like the ICP0 promoter, an ICP27 CRM luciferase reporter construct is cooperatively transactivated by GR and KLF15 (93). Mutating consensus Sp1 binding sites (GGGCGG or the complement CCGCCC) in the respective ICP0 CRM fragments (56, 88) or an ICP27 CRM fragment (87) significantly reduced GR- and KLF15-mediated transactivation. GR and KLF15 form a feed-forward loop where GR stimulates KLF15 expression, and GR subsequently forms a stable complex with KLF15 (Fig. 2C) (94, 95).

### Transactivation of ICP4 CRM sequences by GR and KLF family members

**Fig 3:**
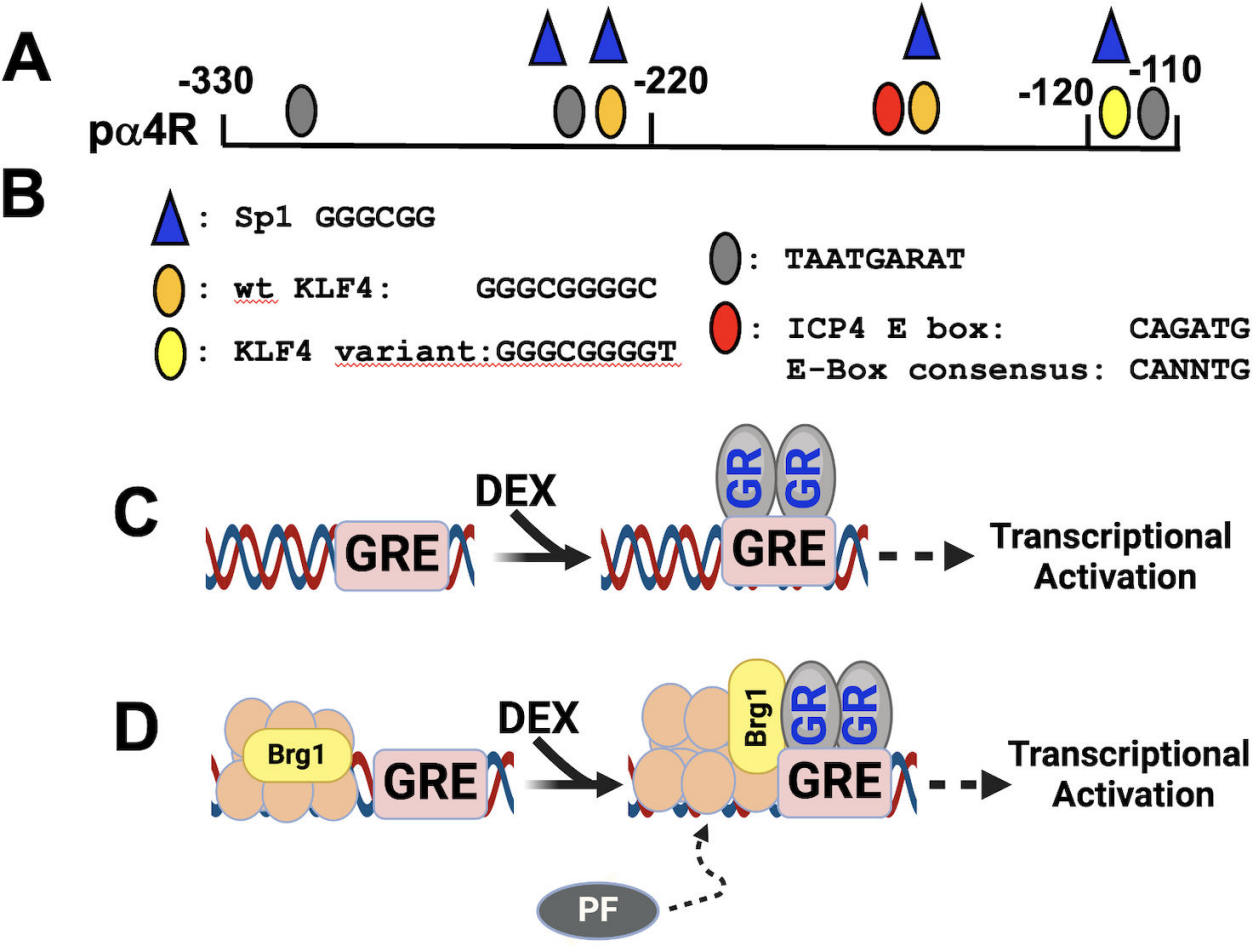
Schematic of ICP4 CRM and the role Brg1 plays during GR-mediated transactivation. (A) The ICP4 CRM fragment (p𝛼4R) was obtained from Dr. Tom Kristie (NIH), and ICP4 CRM sequences were inserted upstream of a minimal promoter in a firefly luciferase reporter plasmid (pGL4.24[luc2/minP]). Denoted nucleotides are relative to the ICP4 transcription initiation site. (B) Position of consensus transcription factor binding sites. (C) Stress, as mimicked by the synthetic corticosteroid DEX, leads to GR binding a consensus GRE, which stimulates transcription. (D) Depiction of a Brg1 complex occupied at or near a GRE. Following DEX treatment, GR binds a GRE, and transcription is increased. Brg1 recruits GR and chromatin remodeling complexes (denoted as the salmon-colored ovals). This complex can subsequently recruit additional pioneer factors (PFs) when GR is bound to a GRE.

An ICP4 CRM construct spanning −330 to −110 (pα4R; Fig. 3A) is synergistically transactivated by GR and KLF4, PLZF, or Slug in Neuro-2A and Vero cells (96). Conversely, GR and KLF15 synergistically transactivate pα4R promoter activity in Vero but not Neuro-2A cells. Two KLF4 binding sites and a variant KLF4 binding site are present in ICP4 CRM sequences (Fig. 3A and B). The consensus KLF4 binding sites, including the variant KLF4 binding site, contain consensus Sp1 binding sites (GGGCGG). GR-, DEX-, and KLF4-mediated transactivation is reduced to basal transcriptional levels when the two consensus KLF4 binding sites are mutated. Mutating the variant KLF4 binding site does not significantly reduce GR- and KLF4-mediated transactivation.

Relative to GR and KLF4-mediated transactivation of the ICP4 CRM, the results were generally the same for GR, PLZF, and DEX-mediated transactivation. PLZF is generally known as a repressor via interactions with the SMRT-Sin3-HDAC-Ncor and Polycomb group complexes, which are typically associated with condensed chromatin, repressive histone marks, and repression of transcription (97–99). EZHT is a histone methyltransferase and a core component of the polycomb repressive complex 2, which initiates and maintains the H3K27me3 repressive epigenetic mark. Surprisingly, interactions between PLZF and EZH2 regulate a group of common genes, which correlates with the active histone mark H3K4me3 and active transcription of these genes (100). The enhancer box (E-Box) and adjacent Sp1/KLF4 binding site are essential for GR-, DEX-, and Slug-mediated transactivation. Slug belongs to the E-box family of transcription factors (101, 102) that positively or negatively regulates transcription. ChIP studies revealed GR, KLF4, Slug, and PLZF occupy ICP4 CRM sequences in transfected Neuro-2A cells and productively infected Vero cells. Since consensus GREs are not present in ICP4 CRM sequences, we suggest GR occupies ICP4 sequences via tethering with KL4, Slug, PLZF, and/or other transcriptional coactivators.

GR (103, 104) and KLF4 (105, 106) are pioneer transcription factors. Pioneer factors, but not “normal” transcription factors, can bind their consensus binding sites when organized as a nucleosome (107). Both KLF4/Sp1 binding sites in the ICP4 enhancer (GGGCGGGGC) match a consensus KLF4 binding site and a preferred nucleosome-depleted KLF4 binding site (105). Notably, GR and KLF4 co-regulate gene expression (108), and KLF4 expression is stimulated by heat stress (109). As expected, GR also interacts with a GRE following DEX treatment (Fig. 3C). Chromatin remodeling serves as a core component of GR-mediated transcriptional regulation. Assembly of the SWI/SNF (SWItch/Sucrose-NonFermentable) remodeling complex and interactions with GR significantly enhance hormone-induced transcription.

Brahma-related gene 1 (Brg1), the central ATPase subunit of the SWI/SNF complex, is associated with at least 10 additional transcriptional coactivators (103, 110–112). Brg1 occupies heterochromatin and recruits GR to a GRE following DEX treatment (Fig. 3D), and silencing Brg1 protein expression impairs GR-mediated gene expression (113). Hence, GR interactions with Brg1 regulate hormone-induced transcription by influencing cohesin binding, enhancer-promoter looping, recruitment of additional pioneer factors, and enhancer RNA expression, reviewed in reference 111.

Since there are no consensus GREs in the IE promoters that were examined, it will be interesting to determine whether Brg1 regulates GR- and KLF4- or KLF15-mediated transactivation. Interactions of two pioneer factors (GR and KLF4) with Brg1 are predicted to increase the incidence of reactivation from latency because HSV-1 exists as silent heterochromatin, and viral transcriptional regulators are not abundantly expressed during latency (6, 7). Additional studies are necessary to delineate the role that pioneer factors play in reactivation from latency.

### Transactivation of a VP16 CRM by GR and Slug

Despite the VP16 promoter being defined as a “leaky-late” promoter in the context of productive infection, several studies concluded VP16 triggers reactivation from latency in a mouse model of infection (114) and neuronal cell-culture latency models, reviewed in reference 115. Downstream of the VP16 start site of transcription, there is a 30 bp GC-rich motif containing Early Growth Response 1 (Egr-1) binding sites and overlapping Sp1 binding sites that are important for heat-stress-induced reactivation from latency (116).

**Fig 4:**
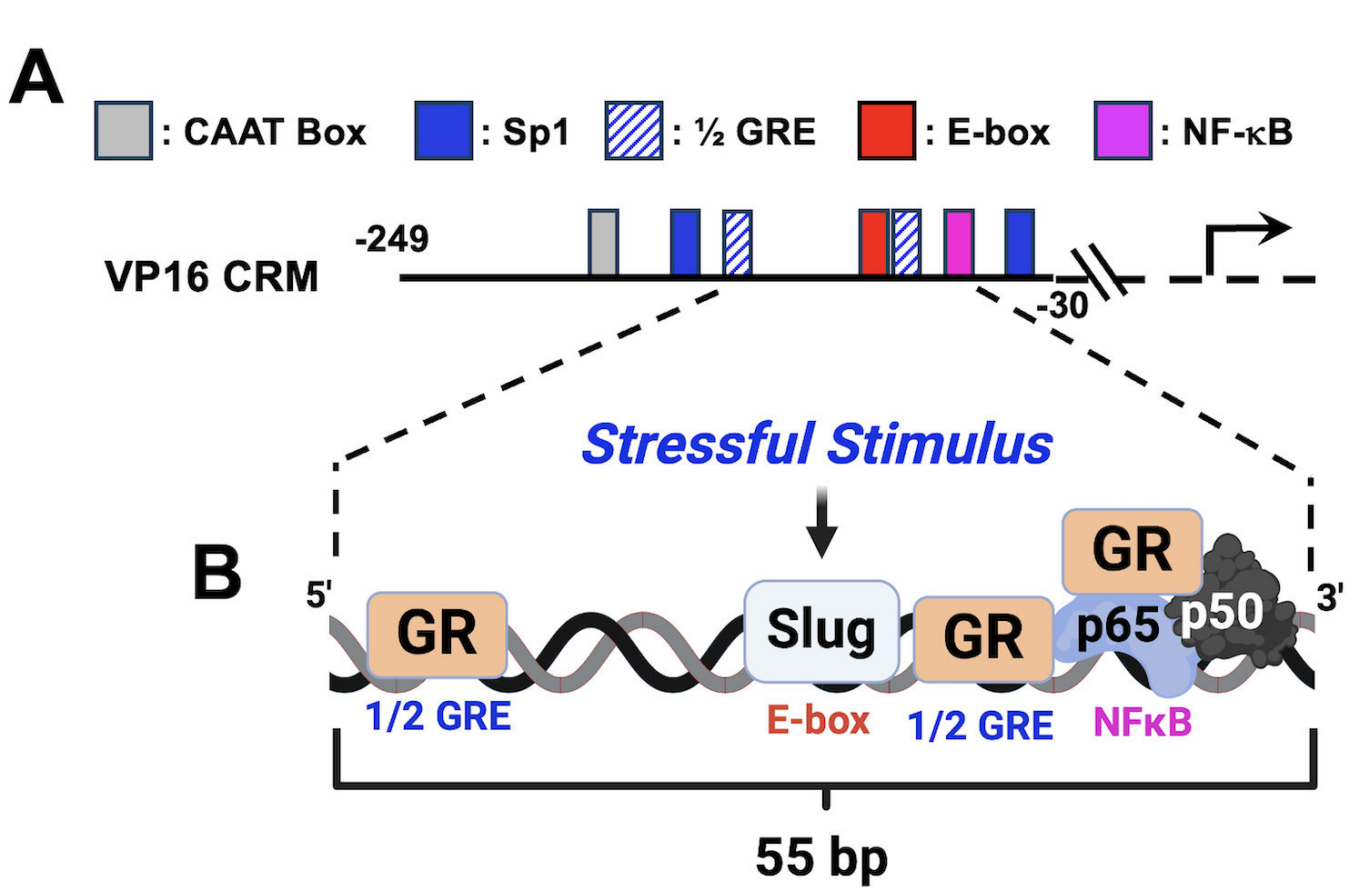
Schematic of HSV-1 VP16 CRM construct. (A) The VP16 CRM spans −249 to −39 bp upstream of the start site of transcription. The arrow marks the start site of VP16 transcription. CRM sequences are upstream of a minimal promoter that drives luciferase expression (pGL4.24[luc2/minP]). Consensus cellular transcription binding sites are denoted. (B) Cartoon depicting how a stressful stimulus triggers binding of cellular transcription factors to VP16 CRM sequences.

VP16 sequences upstream of the TATA box (VP16 CRM) were used to test whether GR and certain stress-induced transcription factors stimulate transcription (Fig. 4A). GR and Slug transactivate the VP16 CRM in an additive fashion; however, DEX had no effect on transactivation (102), suggesting ligand-independent mechanisms mediate transactivation. Notably, Slug is a master regulatory transcription factor for organogenesis, wound healing, and epithelial to mesenchymal transition of cancer cells (101, 117, 118). Mutating the E-box, ½ GREs, or NF-κB binding site, but not Sp1 binding sites, significantly reduced GR- and Slug-mediated promoter activity when compared with the wt VP16 CRM construct. ChIP studies revealed GR and Slug occupy VP16 CRM sequences. Notably, Slug protein expression is detected in more neurons when TG explants from mice are treated with DEX for 3 h (65). Unlike ICP0, ICP4, and ICP27 CRMs, stress-induced KLF family members (KLF4, KLF6, KLF15, or PLZF) do not transactivate the VP16 CRM (102). Although Slug was initially defined as a transcriptional repressor (119), recent studies demonstrated Slug occupancy of an E-box correlates with transcriptional activation of certain promoters (101, 117, 118).

Generally, 1/2 GREs transactivated by a GR monomer contain a 5′-ACA core, which are present in both VP16 1/2 GREs, suggesting that a GR monomer interacts with the VP16 1/2 GREs (Fig. 4B). GR also stably interacts with the p65/p50 heterodimer even when bound to an NF-κB binding site (120), and this interaction generally impairs NF-κB dependent transcription (121). In the context of the VP16 CRM, GR tethering to the p65/p50 heterodimer may play a role in transactivating the VP16 promoter. Recent studies concluded Brg1 activates Slug-dependent transcription (122). Studies focused on whether GR occupancy of the 1/2 GREs and p65/p50 heterodimer that occupy the NF-κB binding site is crucial for GR and Slug-mediated transactivation need to be performed.

## HSV-1 ORIGINS OF REPLICATION AND GG-MEDIATED ACTIVATION

HSV-1 contains two distinct origins of replication (oriL and oriS) (123). The oriL core origin contains a perfect 144 bp palindrome and 20 bp AT-rich sequence within the palindrome. This region is predicted to be unwound during initiation of DNA replication. Adjacent to the AT-rich apex are GRE 1/2 binding sites. Nerve growth factor induces differentiated PC12 neuronal-like cells, and DEX enhances oriL-dependent DNA replication but impairs oriS-dependent DNA replication (123). Conversely, DEX did not enhance oriL-dependent replication in undifferentiated PC12 cells treated with DEX. Introducing point mutations into oriL reduces viral replication and pathogenesis during acute infection and impairs reactivation from latency in mouse models of infection (124). In summary, these studies indicate GR-mediated activation of oriL is important for viral replication in mice and explant-induced reactivation.

## A GR KNOCKIN MUTANT (GR^S229A^) MOUSE STRAIN EXHIBITS REDUCED VIRUS SHEDDING IN FEMALES DURING REACTIVATION FROM LATENCY

*In vivo* studies were necessary to understand the role GR plays during viral replication and reactivation from latency. For these studies, we compared viral replication and reactivation from latency in a mouse strain that contains a serine 229 to alanine mutation in GR (GR^S229A^) versus parental wt C57BL/6 mice. GR^S229A^ mice are healthy, and equal numbers of males and females are born. Mouse GR serine 229, and its human homolog located at GR serine 211 must be phosphorylated for optimal GR-mediated transcriptional activation, reviewed in references 125–127. Virus yields from cornea and conjunctiva of infected GR^S229A^ mice ceased prior to wt mice during acute infection (86). However, viral DNA levels in TG were not significantly different during latency, and similar numbers of TG neurons express GR in GR^S229A^ and wt mice. After DEX treatment, GR^S229A^ mice do not express phosphorylated GR at serine 229, whereas wt mice express phosphorylated GR at serine 229.

DEX-mediated explant-induced reactivation from latency is significantly reduced in female GR^S229A^ mice but not male GR^S229A^ mice or wt C57Bl/6 mice regardless of sex. A lower number of TG neurons from female GR^S229A^ mice express VP16 when compared with male GR^S229A^ mice or wt C57Bl/6 (males or females) 8 h after TG explants are incubated with media containing DEX. This result suggests the number of TG neurons that escape latency was lower in female GR^S229A^ mice relative to age-matched male GR^S229A^ mice or wt C57BL/6 mice. Virus shedding after heat stress-induced reactivation is also lower in TG of female GR^S229A^ mice but not males or wt C57Bl/6 mice (unpublished studies).

Overall, these studies revealed that mutating a single serine→alanine reduced reactivation from latency in female mice. GR knockout mice (126) die at birth due to respiratory failure (128), indicating that GR is an essential gene. HSV-1 replication in primary kidney fibroblasts prepared from GR^S229A^ mice (male or female mice) is significantly lower relative to primary kidney fibroblasts prepared from the parental wt C57BL/6 mice regardless of sex. This result underscores the complexity in TG neurons undergoing reactivation versus productive infection in GR^S229A^ kidney cells.

## CONCLUSIONS

**Fig 5:**
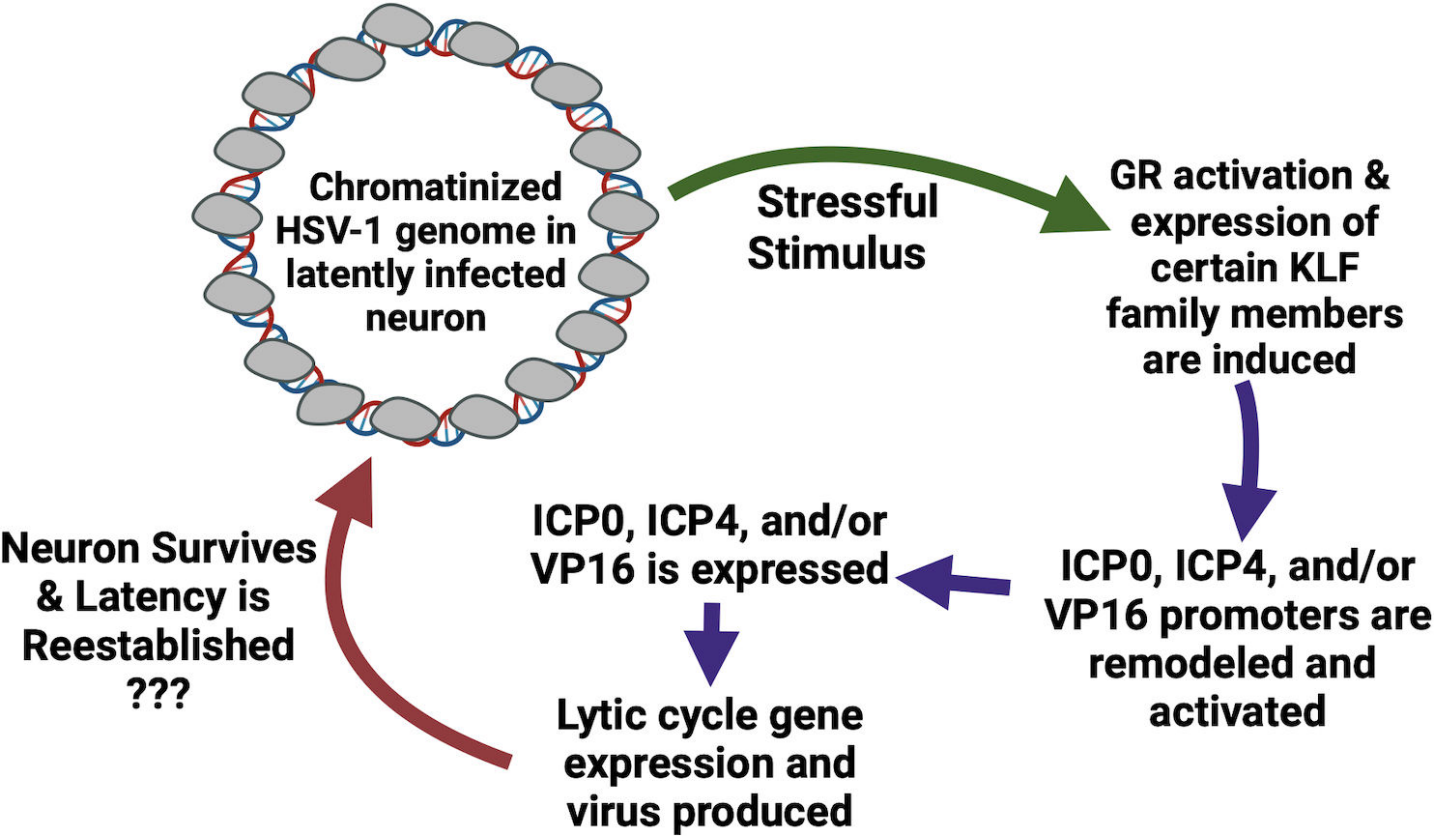
Schematic of hypothetical steps needed to initiate reactivation from latency. For details, see text.

Successful reactivation from latency is a complex series of HSV-1-neuronal interactions that culminate in the production of infectious virus. During latency, the viral genome is organized as silent chromatin (8), and LAT plays a crucial role in maintaining latency (22). Consequently, lytic cycle viral protein expression is not readily detected. Following a stressful stimulus that triggers reactivation from latency, the simplest pathway to reactivation entails at least three events (Fig. 5): (i) VP16, ICP0, and/or ICP4 promoters are remodeled and subsequently activated, (ii) at least one of these viral regulatory proteins must be expressed, and (iii) the lytic cascade of gene expression occurs culminating in production of infectious virus. Since GR is activated by multiple reactivation stimuli (stress, heat stress, or UV light), GR was expected to stimulate the ICP0 promoter plus VP16 and ICP4 CRMs in transient transfection studies. Certain KLF family members, KLF4, KLF15, PLZF, cooperate with GR to activate the ICP0 promoter or ICP4 CRM. Pioneer factors, GR and KLF4 for example, are expected to play a role in reactivation from latency because they can remodel silent chromatin and activate transcription. Finally, GR and KLF15 stimulate viral replication, and GR enhances in explant-induced (57, 86) and heat-stress-induced reactivation (unpublished data). In conclusion, GR and certain stress-induced cellular transcription factors are predicted to drive early stages of reactivation from latency.

This simplistic model may be more complicated because five distinct TG neuronal subtypes and certain subtypes are more permissive for HSV-1 (129), suggesting that viral gene expression is different in specific TG neuronal sub-types during reactivation from latency. Furthermore, HSV-1 establishes latency in autonomic ciliary ganglia neurons (130) and brain (131), and reactivation from latency occurs in mouse models of infection. Hence, VP16, ICP0, or ICP4 expression may be the trigger for reactivation in certain neuronal subtypes. The flexibility of having more than one viral protein that can activate lytic cycle gene expression is crucial for reactivation in the different types of neurons that support HSV-1 latency. Although GR and specific stress-induced reactivation factors are predicted to play important roles during reactivation from latency, other signaling pathways independent of GR can induce reactivation from latency in certain neurons. Regardless of what stimulus initiates reactivation, it is not clear if viral replication occurs during reactivation and whether neurons that reactivate survive and re-establish a latent infection.
